# Detection and Molecular Characterization of Gyrovirus Galga 1 in Chickens in Northern Vietnam Reveals Evidence of Recombination

**DOI:** 10.3390/ani15010067

**Published:** 2024-12-31

**Authors:** Giang Thi Huong Tran, Le Thi My Huynh, Hieu Van Dong, Amonpun Rattanasrisomporn, Autchara Kayan, Dao Anh Tran Bui, Jatuporn Rattanasrisomporn

**Affiliations:** 1 Faculty of Veterinary Medicine, Vietnam National University of Agriculture, Trau Quy Town, Gia lam District, Hanoi 131000, Vietnam; tthgiang@vnua.edu.vn (G.T.H.T.); huynhtmle@vnua.edu.vn (L.T.M.H.); dvhieuvet@vnua.edu.vn (H.V.D.); btadao@vnua.edu.vn (D.A.T.B.); 2 Interdisciplinary of Genetic Engineering and Bioinformatics, Graduate School, Kasetsart University, Bangkok 10900, Thailand; fgraapr@ku.ac.th; 3 Department of Animal Science, Faculty of Agriculture, Kasetsart University, Bangkok 10900, Thailand; fagrark@ku.ac.th; 4 Department of Companion Animal Clinical Science, Faculty of Veterinary Medicine, Kasetsart University, Bangkok 10900, Thailand

**Keywords:** chickens, Gyrovirus galga 1, genetic characterization, Vietnam

## Abstract

In total, 126 tissue-pooled samples were collected from suspected sick chickens showing signs of stunted growth, weakness, and diarrhea in five provinces/cities in Northern Vietnam. The Gyrovirus gala 1 (GyVg1) genome was detected in 26 (20.63%) of the 126 chicken samples based on a polymerase chain reaction assay. The five Vietnamese GyVg1 genomes obtained were all 2.375 nucleotides in length. Among them, the nucleotide identical rates ranged from 94.01% to 100%. Phylogenetic analysis of the complete genomes revealed that the current five GyVg1 strains belonged to different branches at two levels: GyVg1 II (two of five strains) and GyVg1 III (three of five strains). The Vietnamese GyVg1 strains were generically clustered with the Japanese and Chinese strains. Some amino acid substitutions were found in VPs 1, 2, and 3 of the Vietnamese GyVg1 strains. In addition, three and two positive selection sites of VPs 1 and 3 were detected, respectively. A recombination event occurred and generated a recombination Chicken/Vietnam/AGV/VNUA-TN12/2023 strain. These findings indicate the first evidence of GyVg1 viruses circulating in commercial chicken flocks in Vietnam.

## Simple Summary

Gyrovirus galga 1 (GyVg1) strains have been reported to be distributed globally in the chicken production industry. However, there has been no information on the epidemiological and molecular characterization of the GyVg1 genome in Vietnam. The current study was carried out on the detection and characterization of the Vietnamese GyVg1 strains in commercial chickens. The results indicated that GyVg1 strains have been circulating among chickens.

## 1. Introduction

Gyrovirus galga (GyVg) 1 is a member of the genus Gyrovirus of the family *Anelloviridae*. The GyVg1 genome comprises circular, single-stranded DNA [1,2]. Until now, Gyrovirus chicken anemia, one member of the Gyrovirus family, has been reported as an important pathogen causing a contagious disease in chickens. In addition, the structure of the GyVg1 genome is closely related to Gyrovirus chicken anemia and other Gyroviruses. Therefore, it is difficult to differentiate it from other related viral diseases like Gyrovirus chicken anemia and other Gyroviruses. Co-circulation of GyVg1 and other viruses (Newcastle disease virus, Gyrovirus chicken anemia) Hs been reported in chickens and vaccines [3,4,5]. GyVg strains may be associated with clinical manifestations in commercial chickens in the fields [6].

Recently, *Anelloviridae* have been reported to consist of 31 genera containing 155 species. The Gyrovirus genus includes Gyrovirus chicken anemia and 11 recognized species: HgyV/AVG2, GyV3 to GyV11, and GyV13. These 11 viral strains have been reported in some hosts, such as cats, chickens, dogs, ferrets, humans, mice, pigeons, seabirds, snakes, and wild passerine birds [7,8,9,10,11,12,13,14].

The viral genome consists of 2.3 kb encoding the three VPs 1, 2, and 3 proteins [2,15]. In detail, its untranslated region (UTR) length is approximately 490 nucleotides, while three partially overlapping open reading frames (ORFs), including ORF1, ORF2, and ORF3, code for a nonstructural protein VP2, the nonstructural protein VP3, and the viral protein VP1, respectively [15,16]. VP1 contributes to exhibiting viral antigenicity, pathogenicity, and replication [2]. Of the remaining two nonstructural proteins, VP2 plays a role in viral replication and pathogenicity, while VP3 induces apoptosis in infected cells [17].

Currently, GyVg1 infection has been globally reported in some regions of South America (Brazil), Asia (Japan, China), Europe (The Netherlands), and South Africa [6,15,17,18,19,20]. In Japan, GyVg1 was first reported in cryopreserved chicken tissue samples in 1997. The positive rate was 47.5% (19/40) [19]. Based on the complete genome and full-length VP1 gene sequences, the four Japanese GyVg1 belonged to genotypes I and II [19]. The positive rates were lower than that, ranging from 11.69% to 22.46% in China [20]. Zhang et al. reported that the 10 Chinese GyVg1 strains belonged to genotype I and genotype III by analysis of the complete genome [20]. In addition, GyVg1 was considered to infect humans, chickens, and other species [2,7,14,21,22]. Specifically, in chickens, infections by the GyVg1 viruses commence with damage to the brain and declines in mental acuity and weight [5].

In Vietnam, Northern Vietnam is one of the main areas for chicken production (https://www.gso.gov.vn/), where a wide range of infectious diseases may have an affect. Information on GyVg1 infection is limited, with the epidemiology of this virus in chickens and other animals still unknown. Therefore, since GyVg1 has been reported globally in several countries, there is a need to investigate GyVg1 infection in chickens and identify the genetic characterization of GyVg1 strains to gain insight into the GyVg1 infection situation in Vietnam. Consequently, the current study was carried on to determine the molecular characteristics of GyVg1 strains from chickens raised in Northern Vietnam.

## 2. Materials and Methods

### 2.1. Sample Collection

Sample collection was performed from March 2023 to April 2024, and 126 samples were collected from chicken flocks in Hanoi (HN), Thainguyen (TN), Bacninh (BN), Bacgiang (BG), and Haiduong (HD) in Northern Vietnam. Tissue samples of the liver, spleen, lungs, and brain were collected from each suspected sick chicken based on signs of stunted growth, weaknesses, and diarrhea. Tissue samples were homogenated at 10% in phosphate-buffered saline supplemented with gentamicin (10 mg/mL). The homogenates were stored at −80 °C.

### 2.2. Extraction of Total DNA and Polymerase Chain Reaction (PCR)

Extraction of viral DNA from the supernatant of the homogenized samples was performed using Viral Gene-spin™ Viral DNA/RNA Extraction Kits (iNtRON Biotechnology; Seoul, Korea). The extraction protocol followed the manufacturer’s instructions.

For detection, PCR was run using GyVg1-F1/R1 (Table 1), as described elsewhere [18], and Gotaq^TM^ Green Master Mix (Promega, USA). The primers were used to amplify a fragment located in overlapping VP2 and VP3 genes. For sequencing, three primer pairs were previously designed to amplify the 801 or 802 bp, 733 bp, and 981 bp fragments of the GyVg1 genome (Table 1) [15]. The following thermal conditions were applied: a denaturing step at 95 °C for 5 min, then 40 cycles at 95 °C for 30 s, 60 °C for 30 s, and 72 °C for 40 s, and a final extension at 72 °C for 10 min. The PCR product was run in a 1.2% agarose gel and was observed using UV light.

### 2.3. Nucleotide Sequencing and Phylogenetic Analysis

Purification of the PCR products was performed using GeneClean^®^ II Kits (MP Biomedicals; Santa Ana, CA, USA). The GyVg1 genome was sequenced by 1st BASE, Singapore (https://base-asia.com/).

The nucleotide sequence data were compared with other available sequences using GENETYX v.10 software (GENETYX Corp.; Tokyo, Japan) and BLAST (https://blast.ncbi.nlm.nih.gov/Blast.cgi, accessed on 1 September 2024). The Clustal W algorithm supplemented in the BioEdit v.7.2.5 software [23,24] was used to compare the deduced amino acid sequences. The maximum likelihood method with 1000 bootstrap replicates in MEGA version 6.0 software [25] was used to establish phylogenetic trees.

### 2.4. Analysis of Selection Profiles and Recombination Events

The recombination detection program (RDP) version 5.0 was used to analyze the recombination events [26]. Evolutionary selection profiles were examined using Datamonkey (http://www.datamonkey.org/), according to the fast unconstrained Bayesian approximation (FUBAR) method [27].

### 2.5. GenBank Accession Numbers

Deposition of the complete GyVg1 genome sequences obtained in this study was made into GenBank. The accession numbers were PQ154631–PQ154635.

### 2.6. Statistical Analysis

Significant differences in the positive GyVg1 genome detection rate between geographical regions (at the individual and flock levels) were detected using Fisher’s exact test. A significant difference was considered statistically with a value of *p* < 0.05.

## 3. Results

### 3.1. GyVg1 Genome Detection in Field Samples Using PCR

**Figure 1:**
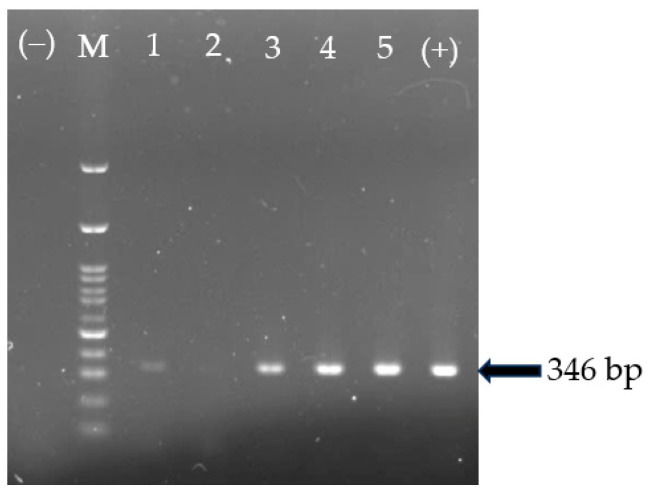
Detection of the GgVg1 genome using PCR. M: 100 bp marker, wells 1–5 were field samples; well (+)/(−) was positive control/negative control.

The GyVg1 genome was detected in 26 out of the 126 (20.63%) samples tested using PCR (Figure 1). In detail, the positive rates for GyVg1 in HN, TN, and BG were 33.33% (12 of 36), 23.80% (10 of 42), and 26.67% (4 of 15), respectively. There was an insignificant difference in the GyVg1-positive percentage in the chickens in the three locations. The GgVg1 genome was not detected in any samples collected from the chickens raised in HD and BN. At the flock level, 7 of 22 (31.81%) farms in the three provinces/cities were positive for the GyVg1 genome in the chickens (Table 2).

### 3.2. Genetic Characterization of the Complete Genome of GyVg1 Strains

Five representative GyVg1-positive samples collected from suspected chicken flocks in HN, TN, and BG were sequenced. The complete genome of five Vietnamese GyVg1 strains were named as Chicken/Vietnam/AGV/VNUA-HN07, -HN25, -TN12, -TN31, -BG09/2023, with shortened names of VNUA-HN07, VNUA-HN25, VNUA-TN12, VNUA-TN31, VNUA-BG09, respectively.

The complete genome sequences (2.375 bp) from five Vietnamese GyVg1 strains were aligned and compared with reference sequences obtained from the GenBank database. The nucleotide sequence identity ranged from 94.01% to 100% among the five Vietnamese GyVg1 strains obtained in the study. The highest nucleotide sequence identity was between VNUA-HN07 and VNUA-HN25 (100%), while the lowest identity was between VNUA-HN07/VNUA-HN25 and VNUA-TN31 (94.01%), as shown in Table 3. In addition, a comparison of the complete genome sequence of five current Vietnamese GyVg1 strains and those available in the GenBank showed the nucleotide sequence identity values of 98.86% (VNUA-TN12 and VNUA-TN31 with HLJ1506-2 [genotype cluster II, accession no. KX708522]), 96.54% to 97.55% (VNUA-HN07, VNUA-HN25, and VNUA-BG09 with N0326-1S [genotype cluster III, accession no. LC716406]) (Table 3).

### 3.3. Phylogenetic Analysis

**Figure 2:**
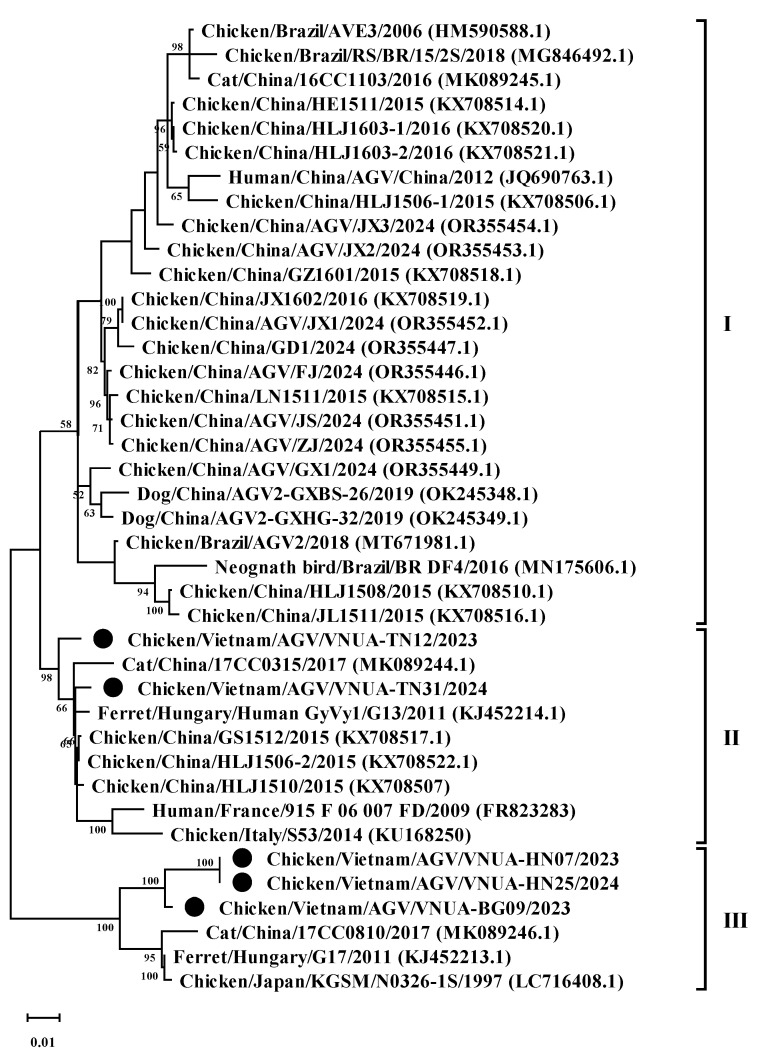
Phylogenetic tree of the complete genome (2.375 bp) sequences of the Vietnamese GyVg1 strains compared to sequences retrieved from GenBank. The phylogenetic tree (1000 bootstrap replicates) was performed using the maximum likelihood method in the MEGA 6 software. Numbers at each branch point demonstrate bootstrap values ≥ 50% in the bootstrap interior branch test. Black circles indicate current Vietnamese GyVg1 strains.

Phylogenetic analysis of the five representative GyVg1 strains based on the complete genome sequence revealed that the five GyVg1 strains belonged to two different clusters, consisting of GyVg1 II (2 of 5 strains) and GyVg1 III (3 of 5 strains), as shown in Figure 2. In detail, the current VNUA-TN12 and VNUA-TN31 strains clustered together in subgenotype cluster II (Figure 2). They closely related to that of the previous Chinese GyVg1 reported in 2015–2017 (accession nos. KX708522.1, KX708507, and MK089244.1). In addition, based on the current results, the three GyVg1 strains (VNUA-07, VNUA-25, and VNUA-BG09) were within genotype cluster III (Figure 2). They shared closely with the Chinese strain reported in 2015 (accession no. KX708522) and the Japanese GyVg1 strains reported in 1997 (accession no. LC716408).

### 3.4. Analyses of Protein Profiles

**Figure 3:**
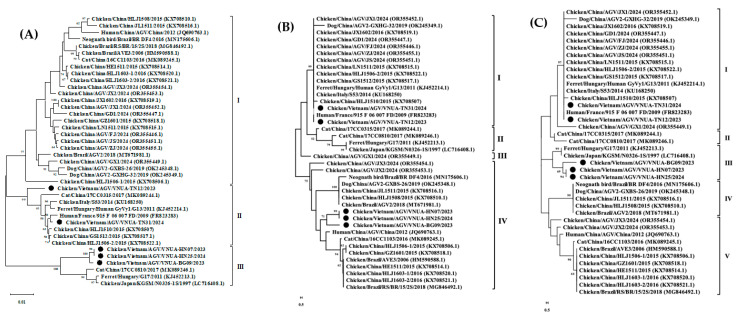
Phylogenetic trees of VPs (**A**) 1 (460 amino acids), (**B**) 2 (125 amino acids), and (**C**) 3 (125 amino acids) proteins of Vietnamese GyVg1 strains compared with those sequences retrieved from GenBank. The maximum likelihood method in MEGA6 was used to build the phylogenetic trees with 1000 bootstrap replicates. Black filled circles show Vietnamese GyVg1 strains.

Analysis of predicted amino acid of VP1 protein: The VP1 protein sequences of the five current GyVg1 strains were 460 amino acids in length. There were three major groups in the phylogenetic analysis of the VP1 protein amino acid sequence (460 amino acids). In the present results, the five GyVg1 strains belonged to clusters II and III (Figure 3A). In detail, VNUA-TN12 and VNUA-TN31 were clustered in group II, while VNUA-HN07, VNUA-HN25, and VNUA-BG09 belonged to group III.

The analysis of the deduced amino acid sequences of the five GyVg1 strains obtained indicated no amino acid change at the three motifs amino acid sequence at positions 325–329 (AALS), 363–369 (RRWLTLV), and 412–415 (KAMA). In addition, there were some amino acid substitutions in the VP1 protein: specifically, Q to S, V to Q, G to Q, and R to K amino acid substitutions at positions 145, 154, 288, 293, and 314, respectively, in the VP1 protein of the three strains VNUA-HN07, VNUA-HN25, and VNUA-BG09 (Table 4). Furthermore, an amino acid change at position 135 (G to S) was observed in the VP1 protein of the VNUA-HN07 and VNUA-HN25 strains. There was an amino acid substitution at position 408 (G to R) in the VP1 of VNUA-31 (Table 4).

Analysis of VP2 protein: The VP2 sequences of the five current GyVg1 strains obtained in the study were 231 amino acids in length. The phylogenetic tree of the VP2 amino acid sequence was divided into four major groups (I–IV). The current GyVg1 strains in the study belonged to genetic clusters I and IV (Figure 3B). Specifically, VNUA-TN12 and VNUA-TN31 were clustered in group I, while the VNUA-HN07, VNUA-HN25, and VNUA-BG09 belonged to group IV.

The sequence of the phosphatase motif WLRQCARSHDEICTCGRWRSH at position 95–115 was highly conserved in the five current GyVg1 strains. In addition, there were amino acid substitutions at positions 134, 141, 156–158, 165, 174–175, and 179 in the VP2 proteins of the five current GyVg1 strains (Table 5). Notably, there was a unique amino acid substitution at position 134 (A to T) in the VP2 protein of VNUA-BG09. Furthermore, the five Vietnamese GyVg1 strains were amino acid deficient at position 162 in their VP2 proteins.

Analysis of VP3 protein: The VP3 amino acid sequences of the five Vietnamese GyVg1 strains were 125 amino acids in length. The phylogenetic tree of the VP3 amino acid sequence was divided into five genetic clusters (I–V). Based on the results of the current study, VNUA-TN12 and VNUA-TN31 were classified into genetic cluster I, while VNUA-HN07, VNUA-HN25, and VNUA-BG09 were grouped in cluster III (Figure 3C).

Based on the analysis of the VP3 amino acid sequences, there were changes in amino acid at positions 9, 14, 28, 99, 104, 115, 122, and 125 in the VP3 proteins of VNUA-HN07, VNUA-HN25, and VNUA-BG09 (Table 6). In addition, amino acid substitutions at positions 7 and 81 were first found in the VP3 protein of the VNUA-BG09 strain. The five GyVg1 strains in this study had R insertion in the VP3 amino acid sequence at position 122.

### 3.5. Selection Profiles Among Current Vietnamese GyVg1 Sequences

Regarding the analysis of natural selection, the three sites 145, 288, and 293 in the VP1 genes of all the current GyVg1 strains were considered as positive selections. In addition, positive selection was determined at two positions (81 and 99) of the VP3 protein (Table 7). Some sites were negative selections in VP1, VP2, and VP3 of the five current GyVg1 strains (Supplemental Table S1).

### 3.6. Analysis of Recombination Events

**Figure 4:**
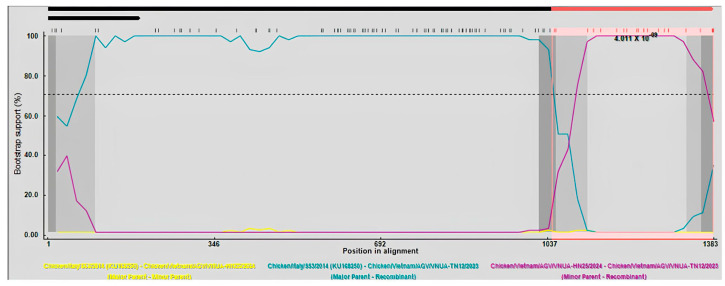
BootScan analysis was used to identify recombination events based on the full-length VP1 gene sequences of Vietnamese GyVg1 strains. Algorithms implemented in the RDP program were used with 1000 bootstrap replicates. Yellow, green, and violet lines indicates as minor parent, major parent, and recombination strains, respectively.

Recombination analysis of the full-length VP1 gene sequences indicated that a recombination event occurred to generate the VNUA-TN12 recombination strain. The Chicken/Italy/S53/2014 and VNUA-HN25 strains were the major and minor parents, respectively. This putative recombination event was determined by six out of the nine methods using RDP4 (Table 8). Nucleotides at the 1021 and 1121 positions of the VP1 gene were identified as two breakpoints (Figure 4).

## 4. Discussion

GyVg1 was considered a newly discovered Gyrovirus in 2011 [21], even though the presence of this virus was reported in Brazil in 2008 [2]. GyVg1 has been reported in poultry production globally [8,9,12,17]. The current study was the first to report GyVg1 in commercial broiler chickens in Northern Vietnam.

In this study, the GyVg1 infection was identified in 20.63% of the chicken samples in the five provinces/cities in 2023–2024. This rate was considered higher than the percentage of GyVg1 infection in chickens farmed in China, which reported about 12.28% from 2015 to 2016 [28], but less than that of 60.32% in the southern Netherlands or 84.41% in Brazil. This further supports the hypothesis that geographical differences and the environment may result in percentage differences [6]. Although, the pathogenicity of GyVg1 has not been clearly described, notably, all the current samples were collected in chickens showing weakness, stunted growth, and diarrhea. Therefore, the pathogenicity of GyVg1 needs to be further explored.

The complete genomes obtained in the current study were each 2375 nucleotides in length, while other GyVg1 sequences available in GenBank were approximately 2380 bp in length, with the difference being due to the varying length of the G and poly C regions [15]. Additionally, all five current GyVg1 sequences had a serine (S) deletion at position 162 in the VP2 protein, while some novel GyVg1 sequences had an S insertion at this site of the VP2 protein [15]. The genetic characterization of the GyVg1 strains in Northern Vietnam was investigated based on the complete genome sequence of five GyVg1 strains obtained in the study. Based on the results, the five current strains had a >90% nucleotide identical rate at the nucleotide sequence level with other available GyVg1 sequences in the GenBank. This is consistent with other reports that the genome of current GyVg1 strains from chickens in Vietnam is highly conserved [19,29].

The phylogenetic analysis of the amino acid and nucleotide sequences did not perfectly match with each other [15], perhaps due to synonymous mutations. In the current study, the Vietnamese GyVg1 strains were also divided into several genotypes when comparing the VPs 1, 2, and 3 proteins. This indicated that various genotypes have been prevalent in Vietnamese chicken flocks in the 21st century. Furthermore, the sequences of the VPs 1, 2, and 3 proteins of the five Vietnamese GyVg1 strains remained well conserved [15].

All VP1 protein sequences have been reported as being 460 amino acids in length [15,19], including those detected in Vietnam. The function of the VP1 protein may be mainly to induce a host immune response to Gyroviruses [15]. Although there was a series of amino acid substitutions in the VP1 protein identified in the study, the function of the VP1 protein of GyVg1 has not been well understood. It has been suggested that the hypervariable region is detected in positions 228 to 314 of the VP1 protein [19,20]. One amino acid substitution at position 288 was observed in the hypervariable region of all five current GyVg1 strains. In addition, the positive sites were reported with amino acids at positions 36, 288, and 293 [29]. Based on the current results, there were three amino acid-positive sites (including 288 and 293) in the VP1 protein of all five Vietnamese GyVg1 strains. This finding further confirms the evolutionary advantages of GyVg1 strains. Furthermore, these positive selection positions are speculated to help GyVg1 evade immune responses in animals.

The VP2 protein of GyVg1 was mostly conserved among the five current GyVg1 strains. In addition, the role of VP2 protein phosphatase activity was important for Gyrovirus chicken anemia [30]. Furthermore, some amino acid substitutions were found in the current Vietnamese GyVg1 strains. The importance of the phosphatase motif in GyVg1 VP2 regarding its effect on GyVg1 requires further investigation, although GyVg1 VP2 may have the same function as that of Gyrovirus chicken anemia.

The GyVg1 VP3 protein has been reported to induce apoptosis of tumor cells [16]. The VP3 protein may play a critical role in inducing apoptosis in infected cells [31,32]. The domains proposed were a putative leucine-rich domain at positions 38 to 51, a putative nuclear export signal domain at positions 102 to 110, and other sites at 84–88 and 116–124 [16]. In the current study, some amino acid substitutions were found in the NES and NLS2 domains and other positions in the VP3 of the GyVg1 strains. These might affect the function of VP3 GyVg1. Furthermore, positive selection sites were detected in the VP3 protein of the current Vietnamese VP3, which may provide evolutionary advantages to GyVg1. Notably, GyVg1 and Gyrovirus chicken anemia have contaminated various vaccines [4].

Other studies have reported evidence of recombination events among Gyroviruses [3,29,33,34]. This study’s results are evidence of a recombination event in Vietnamese GyVg1 strains in the VP1 protein. This is strongly consistent with other findings that the genomes of GyVg1 have some recombination regions, including the VP1 protein and others [29].

## 5. Conclusions

Overall, this investigation reported the prevalence of GyVg1 in commercial chickens in Vietnam from 2023 to 2024. The data provided a comprehensive genetic characterization of GyVg1 circulating among chickens in Northern Vietnam. In addition, further studies should be conducted to investigate the evolution of GyVg1.

## Figures and Tables

**Table 1:** Primers used in this study.

Name	Primer Sequence (5′–3′)	Fragment Size (bp)	Reference	Purpose
GyVg-F1	CGTGTCCGCCAGCAGAAACGAC	346	[15]	Detection
GyVg-R1	GGTAGAAGCCAAAGCGTCCACGA			
QC1F	ATTTCCTAGCACTCAAAAACCCAT T	802	[11]	Nucleotide sequencing
QC1R	TCTGGGCGTGCTCAATTCTGATT			
QC2F	TCACAGCCAATCAGAATTGAGCACG	733		
QC2R	TTCTACGCGCATATCGAAATTTACC			
QC3F	TATTCCCGGAGGGGTAAATTTCGAT	981		
QC3R	CCCCTGTCCCCGTGATGGAATGTTT			

**Table 2:** Identification of GyVg1 DNA in chickens in different locations of Northern Vietnam.

Location	No. of Sample Collection	No. of Positive Samples (%)	No. of Flocks	No. of Positive Flocks/(%)
Hanoi (HN)	36	12 (33.33) ^a^	6	3 (50.00)
Haiduong (HD)	22	0 (0) ^b^	4	0 (0)
Thainguyen (TN)	42	10 (2.80) ^a^	5	3 (60.00)
Bacgiang (BG)	15	4 (26.67) ^a^	3	1 (33.33)
Bacninh (BN)	11	0 (0) ^b^	4	0 (0)
Total	126	26/(20.63)	22	7/(31.81)

^a, b^ indicated groups significantly (*p* < 0.05) different.

**Table 3:** Nucleotide sequence identities of complete genome of Vietnamese GyVg1 strains with representative strains.

Cluster	Strain Name (GenBank Accession Number)	Number of Strains/Nucleotide Sequence Identity (%)										
		1	2	3	4	5	6	7	8	9	10	11
I	1. AGV/JS (OR355451)											
I	2. HE1511 (KX708514)	98.02										
II	3. GS1512 (KX708517)	97.68	96.08									
II	4. HLJ1506-2 (KX708522)	97.72	96.12	99.95								
III	5. G17 (KJ452213)	95.20	95.53	95.49	95.45							
III	6. N0326-1S (LC716408)	95.12	95.46	95.33	95.29	99.82						
	7. VNUA-HN07	93.07	94.08	93.89	93.85	96.54	96.40					
	8. VNUA-HN25	93.72	94.48	93.89	93.85	96.54	96.40	**100**				
	9. VNUA-TN12	97.68	96.29	98.82	98.86	95.49	95.20	**94.73**	**94.73**			
	10. VNUA-TN31	97.51	95.96	99.45	99.49	95.32	95.08	**94.01**	**94.01**	**99.19**		
	11. VNUA-BG09	94.90	95.70	95.15	95.11	97.55	97.47	**98.35**	**98.35**	**95.91**	**95.19**	

Underlining indicates the highest identity of the completed genome of Vietnamese GyVg1 strains with representative strains. The nucleotide identity of the completed genome among the Vietnamese GyVg1 strains is in bold.

**Table 4:** Amino acid profile in VP1 proteins of Vietnamese GyVg1 strains and other representative strains from GenBank.

Virus Strain	VP1 Protein							
	36	145	154	288–314	325–329	363–369	408	412–415
GyVg1 * (I–III)	G/S	Q	A/S	V^288^…G^293^…R^314^	FAALS	RRWLTLV	G	KAMA
VNUA-HN07	S	S	S	Q^288^…Q^293^…K^314^	.	.	.	.
VNUA-HN25	S	S	S	Q^288^…Q^293^…K^314^	.	.	.	.
VNUA-TN12	G	. ^a^	A	.	.	.	.	.
VNUA-TN31	G	.	A	.	.	.	R	.
VNUA-BG09	S	S	S	Q^288^…Q^293^…K^314^	.	.	.	.

* Representative GyVg1 belonging to clusters I–III from GenBank. ^a^ Same as representative GyVg1 sequence.

**Table 5:** Amino acid profile in VP2 protein of Vietnamese GyVg1 and other representative GyVg1 strains.

Virus Strain	VP2 Protein								
	95–115	134	141	156–158	162	165	167	174–175	179
GyVg1 * (I–III)	WLRQCARSHDEICTCGRWRSH	A	K/Q	GKR	_/S	A	T	EE	A
VNUA-HN07	. ^a^	.	Q	RRG	_	T	S	DD	V
VNUA-HN25	.	.	Q	RRG	_	T	S	DD	V
VNUA-TN12	.	.	K	.	_	.	.	.	.
VNUA-TN31	.	.	K	.	_	.	.	.	.
VNUA-BG09	.	T	Q	RRG	_	T	S	DD	V

* Representative GyVg1 belonging to clusters I–III from GenBank. ^a^ Same as representative GyVg1 sequence. “_” deletion of amino acid residues at the location.

**Table 6:** Amino acid substitutions in VP3 protein of Vietnamese GyVg1 isolates and other representative GyVg1 strains.

Virus Strain	VP3 Protein									
	7	9	14	28	81	99	104	115	122	125
GyVg1 * (I–III)	R	R	Q	S	L	A	Q	N	K	L
VNUA-HN07	. ^a^	Q	R	C	S	N	R	E	R	V
VNUA-HN25	.	Q	R	C	S	N	R	E	R	V
VNUA-TN12	.	.	.	.	.	.	.	.	.	.
VNUA-TN31	.	.	.	.	.	.	.	.	.	.
VNUA-BG09	H	Q	R	C	S	S	R	E	R	V

* Representative GyVg1 belonging to clusters I–III from GenBank. ^a^ Same as the representative GyVg1 sequence.

**Table 7:** Positive selections in VP1 and VP3 protein sequences of Vietnamese GyVg1 strains.

Protein	Amino Acid Position	a	b	b − a	Prob [a > b]	Prob [a < b]	Bayes Index [a < b]
VP1	145	3.03	23.97	20.94	0.06	0.91	18.46
	288	2.48	25.19	22.71	0.04	0.93	24.55
	293	2.81	23.89	21.09	0.05	0.92	20.17
VP3	81	1.85	23.18	21.33	0.07	0.90	10.57
	99	3.60	32.39	28.79	0.03	0.94	18.67

a: indicates posterior synonymous substitution rate at a site. b: indicates posterior non-synonymous substitution rate at a site. Prob [a < b] ≥ 0.9: posterior probability of positive selection at a site.

**Table 8:** Recombination statistics of Chicken/Vietnam/AGV/VNUA-TN12/2023 using RDP4.

No.	Method	Recombination *p*-Value
1	RDP	-
2	GENECONV	2.85 × 10^−8^
3	BootScan	8.73 × 10^−10^
4	MaxChi	6.73 × 10^−6^
5	Chimaera	9.74 × 10^−5^
6	SiScan	1.59 × 10^−7^
7	PhylPro	1.80 × 10^−9^
8	LARD	-
9	3Seq	-

*p*-value < 0.05: Recombination events occurred.

## Data Availability

The data presented in this study are available within the article. Raw data supporting this study are available from the corresponding author.

## Supplementary Materials

The following supporting information can be downloaded at: https://www.mdpi.com/article/10.3390/ani15010067/s1, Table S1: Negative selection in VP1. VP2. and VP3 protein sequences of Vietnamese GyVg1 strains.
