# Supplementary material for: Detection and Molecular Characterization of Gyrovirus Galga 1 in Chickens in Northern Vietnam Reveals Evidence of Recombination
Source: Animals (Basel). 2024 Dec 31;15(1):67. doi: 10.3390/ani15010067 (PMC11718778; doi:10.3390/ani15010067)
Supplement: Supplementary file 1 [file animals-15-00067-s001.zip › animals-3331212-supplementary.pdf]

Supplemental Table S1. Negative selection in VP1. VP2. and VP3 protein sequences of Vietnamese GyVg1 strains

Protein	amino acid position	a	b	b-a	Prob [a > b]	Prob [a < b]	Bayes Index [a < b]
	4	16.76	0.76	-15.99	0.92	0.06	0.12
	6	18.36	1.24	-17.13	0.92	0.07	0.13
	7	18.36	1.24	-17.13	0.92	0.07	0.13
	10	14.98	0.69	-14.29	0.92	0.06	0.12
	34	17.32	0.92	-16.40	0.92	0.06	0.12
	75	18.47	0.72	-17.74	0.94	0.05	0.09
	77	19.72	0.59	-19.13	0.95	0.04	0.08
	88	17.08	0.72	-16.36	0.93	0.06	0.10
	92	19.72	0.65	-19.07	0.95	0.04	0.08
	93	21.06	0.71	-20.35	0.95	0.04	0.07
	99	15.88	0.74	-15.14	0.92	0.06	0.12
	105	23.69	0.72	-22.97	0.96	0.03	0.05
	117	18.07	0.72	-17.35	0.94	0.05	0.10
	131	19.72	0.65	-19.07	0.95	0.04	0.08
	147	23.00	0.69	-22.32	0.95	0.04	0.07
	159	33.87	0.69	-33.18	1.00	0.00	0.00
	190	16.11	0.69	-15.42	0.93	0.06	0.11
	191	19.44	0.72	-18.72	0.94	0.05	0.09
	199	17.98	0.88	-17.09	0.93	0.06	0.11
	201	18.99	0.77	-18.22	0.94	0.05	0.08
	203	16.06	0.71	-15.35	0.93	0.06	0.11
VP1	208	19.72	0.59	-19.13	0.95	0.04	0.08
	209	16.85	0.73	-16.11	0.93	0.06	0.11
	210	19.12	0.65	-18.47	0.94	0.05	0.09
	212	19.88	0.72	-19.16	0.94	0.05	0.08
	213	15.15	0.68	-14.47	0.92	0.06	0.12
	219	23.38	0.72	-22.66	0.96	0.03	0.05
	220	17.08	0.72	-16.36	0.93	0.05	0.10
	222	16.11	0.72	-15.39	0.93	0.06	0.11
	225	14.13	0.69	-13.44	0.92	0.07	0.13
	226	19.72	0.59	-19.13	0.95	0.04	0.08
	227	20.23	0.69	-19.53	0.94	0.05	0.09
	228	18.06	0.72	-17.34	0.94	0.05	0.10
	232	14.12	0.77	-13.35	0.91	0.07	0.14
	234	16.09	0.68	-15.41	0.93	0.06	0.11
	243	19.72	0.66	-19.06	0.95	0.04	0.08
	253	16.11	0.69	-15.42	0.93	0.06	0.11
	256	19.75	0.64	-19.12	0.95	0.04	0.08
	261	19.88	0.72	-19.16	0.94	0.05	0.08
	262	14.13	0.68	-13.46	0.92	0.07	0.13
	264	19.45	0.79	-18.66	0.94	0.05	0.09
	267	19.72	0.65	-19.07	0.95	0.04	0.08
	273	19.53	0.73	-18.80	0.94	0.04	0.08
	274	18.07	0.72	-17.35	0.94	0.05	0.10

	276	19.45	0.71	-18.74	0.94	0.05	0.09
	277	19.88	0.78	-19.10	0.94	0.05	0.09
	280	21.06	0.72	-20.34	0.95	0.04	0.07
	285	19.72	0.66	-19.06	0.94	0.04	0.08
	290	18.06	0.72	-17.34	0.94	0.05	0.10
	294	20.74	0.86	-19.88	0.94	0.04	0.08
	296	15.15	0.65	-14.50	0.92	0.06	0.12
	297	19.88	0.78	-19.10	0.94	0.05	0.09
	299	19.50	0.71	-18.79	0.94	0.04	0.08
	316	19.12	0.59	-18.53	0.95	0.04	0.08
	317	19.16	0.64	-18.51	0.94	0.04	0.08
	320	17.08	0.72	-16.36	0.93	0.06	0.10
	321	19.15	0.63	-18.51	0.94	0.04	0.08
	329	14.13	0.65	-13.49	0.92	0.07	0.13
	338	18.56	0.73	-17.84	0.94	0.05	0.09
	340	17.08	0.72	-16.36	0.93	0.05	0.10
	343	14.13	0.68	-13.45	0.92	0.07	0.13
	344	19.29	0.69	-18.60	0.94	0.05	0.09
	348	29.19	0.69	-28.49	0.99	0.01	0.01
	351	39.07	0.69	-38.38	1.00	0.00	0.00
	352	22.39	0.62	-21.77	0.95	0.04	0.07
	374	23.68	0.68	-23.01	0.96	0.03	0.06
	378	22.10	0.63	-21.47	0.95	0.04	0.07
	383	19.51	0.70	-18.81	0.94	0.05	0.09
	394	22.08	0.58	-21.50	0.95	0.04	0.07
	399	23.74	0.64	-23.10	0.96	0.03	0.06
	401	20.76	0.65	-20.11	0.95	0.04	0.08
	413	19.41	0.70	-18.71	0.94	0.05	0.09
	415	16.53	0.69	-15.84	0.92	0.06	0.12
	416	15.36	0.77	-14.59	0.91	0.07	0.13
	423	18.44	0.69	-17.76	0.94	0.05	0.10
	427	19.40	0.65	-18.75	0.94	0.05	0.09
	430	17.15	0.76	-16.39	0.93	0.06	0.11
	442	22.09	0.64	-21.46	0.95	0.04	0.07
	452	22.09	0.63	-21.47	0.95	0.04	0.07
VP2	113	20.20	1.11	-19.09	0.91	0.07	0.10
	123	26.50	1.61	-24.90	0.93	0.05	0.07
	164	21.80	1.10	-20.70	0.92	0.06	0.09
	172	30.13	1.09	-29.04	0.96	0.03	0.04
	202	25.53	1.28	-24.26	0.93	0.05	0.07
	204	23.27	1.18	-22.09	0.92	0.06	0.08
VP3	70	22.92	0.96	-21.96	0.92	0.07	0.08
